# Internalized stigma and associated factors among people with mental illness at University of Gondar Comprehensive Specialized Hospital, Northwest, Ethiopia, 2021

**DOI:** 10.1186/s13033-022-00567-2

**Published:** 2022-12-31

**Authors:** Lamesa Melese Sori, Faisel Dula Sema, Masho Tigabe Tekle

**Affiliations:** 1 Department of Psychiatry, University of Gondar Comprehensive Specialized Hospital, P. Box: 196, Gondar, Ethiopia; 2 Department of Clinical Pharmacy, School of Pharmacy, College of Medicine and Health Sciences, University of Gondar, P. Box: 196, Gondar, Ethiopia

**Keywords:** Prevalence, Internalized stigma, Factors, Mental illness

## Abstract

**Background:**

Internalized stigma has been found to be high among people with mental illness (PWMI) and it results in poor treatment outcome, increased disability and high economic burden. So, this study was designed to determine the prevalence and associated factors of high internalized stigma among PWMI attending psychiatric follow-up at University of Gondar Comprehensive Specialized Hospital, Northwest, Ethiopia, 2021.

**Methods:**

A cross-sectional study was conducted among PWMI (n = 365), and internalized stigma was measured by using internalized stigma of mental illness 29 (ISMI-29) scale. The data was entered in to EPI DATA software (4.6.0.2) and analyzed by Statistical Package for Social Sciences version 20. A binary logistic regression was used to identify factors associated with internalized stigma and reported with 95% confidence interval (CI). P-value < 0.05 was considered as statistically significant.

**Results:**

The prevalence of high internalized stigma was found to be 27.9% (95% CI 23.1–32.6). A male gender (AOR = 0.332; 95% CI 0.175–0.629), occupation, specifically government employee (AOR = 0.309; 95% CI 0.118–0.809), life time substance use (AOR = 3.561; 95% CI 1.867–6.793), low self-esteem (AOR = 8.313; 95% CI 3.641–18.977), and history of hospitalization (AOR = 4.236; 95% CI 1.875, 9.570) were factors significantly associated with higher internalized stigma.

**Conclusion:**

The result of this study showed that there was an intermediate prevalence of high internalized stigma among PWMI at University of Gondar Comprehensive Specialized Hospital. The hospital needs to take immediate action to fight internalized stigma by focusing on females, people with low self-esteem, individuals with history of lifetime substance use, and people who have history of hospital admission.

**Supplementary Information:**

The online version contains supplementary material available at 10.1186/s13033-022-00567-2.

## Background

Stigma is defined as ‘a mark of shame, disgrace, or disapproval that results in an individual being rejected, discriminated against, and excluded from participating in a number of different areas of society’ [1]. Evidences described that there are multiple types of mental health related stigma including internalized stigma (self-stigma), public stigma, professional stigma, and institutional stigma [2–5]. Stigma is a major problem of adults with mental illness (MI) which can lead to patients’ impoverishment, social marginalization, worsening of the disease, low quality of life, decreased health-seeking behavior, poor adherence to medication, and have a negative impact on socio-economic well-being [6–11].

Internalized stigma is the process by which individuals with MI apply negative stereotypes to themselves, expect to be rejected by others, and feel alienated from society [7]. PWMI are facing double problems: their illness and the stigma [12]. Internalized-stigma associated with MI is a global public health problem affecting help seeking behavior, social interactions, adherence to prescribed medications, self-esteem, hope, health-care, productivity, and acceptance among others [13–19]. Further, it has been linked to exacerbation of MI, poor treatment outcomes, increased disability, high economic burden, poor quality of life, increased suicidal attempt, and mortality [13, 17, 18, 20, 21]. A cross-sectional study in Egypt showed that internalized stigma was significantly associated with high suicidal risk of schizophrenia patients [22]. A study in Saudi Arabia revealed that there was a significant positive relationship between patients’ perceived stress and their feelings of internalized stigma [23].

Several studies described that the prevalence of internalized stigma among PWMI is high, ranging from 8.1% to 54.44% [13, 16, 17, 19, 20, 24–31]. A cross-sectional study conducted in Nepal reported that the prevalence of internalized stigma among mentally ill people was 54.44% [17]. Another study conducted in Singapore found that the score of moderate to high internalized stigma was 43.6% [16].

In Ethiopia, despite very few studies have been conducted on internalized stigma and its associated factors, evidences described that internalized stigma is highly prevalent among PWMI. A previous studies conducted in Jimma University Specialized Hospital and Dilla university referral hospital reported that the prevalence of internalized stigma among PWMI was 25.12% [13] and 32.1% [30], respectively.

As to the several studies, internalized stigma among PWMI is affected by a variety of individual patient's characteristics including socio-demographic variables, psychosocial, substance, clinical, and treatment related factors. Moreover, these studies indicated that unemployment, family history of MI, having previous hospitalization due to MI, relapse after starting the treatment, being a female, single in marital status, non-compliance to medication treatment, rural residence, having prominent psychotic symptoms, history of suicidal attempt, poor social support, being diagnosed with schizophrenia, longer duration of illness, and low self-esteem were significantly associated with high internalized stigma [13, 17, 24, 27–30, 32, 33].

In combating the negative influence of internalized stigma among PWMI, identification and targeting the potential risk factors for its high prevalence is the starting point. Through generating scientific evidence of risk factors of high internalized stigma, this study may provide data for clinicians on which adjustable risk factors they should target in reducing the internalized stigma of their patients. Moreover, the finding of this study might assist in designing and implementation of strategies such as improving the self-esteem of PWMI, empowerment through health education, and improving their help seeking behavior) which are essential for reducing internalized stigma among PWMI. In Ethiopia, there is a widespread mental illness and associated deep-rooted stigma towards PWMI. Although some effort tried to reduce stigma against PWMI, stigmatization, and discrimination towards PWMI, it is still persistent. A study in the capital city of Ethiopia, Addis Ababa reported that almost all schizophrenia patients encountered internalized stigma [34]. To the best of the authors’ literature search, a very few scientific evidences are found in Ethiopia regarding prevalence and determinants of internalized stigma among PWMI [13, 30, 31]. As understanding the prevalence and its associated factors of internalized stigma related to mental illness is very crucial the finding of this study will assist in designing prevention strategies and giving direction to minimize deleterious effect of internalized stigma on lives of PWMI. In addition, the study will have a great relevance for stakeholders to tackle the problem because understanding the magnitude of the problem and modifiable risk factors is imperative. Further, this study will provide baseline information for similar studies that are going to be conducted in the future. Therefore, this study was conducted to determine the prevalence of internalized stigma and associated factors among PWMI attending psychiatric follow-up at University of Gondar Comprehensive Specialized Hospital (UOGCSH), Northwest, Ethiopia, 2021.

## Methods

### Study design, area and period

This institutional-based cross-sectional study was conducted in Psychiatry clinic of University of Gondar Comprehensive Specialized Hospital from February 1, 2021 to July 1, 2021 which is found in Gondar town, 738 km away from the capital city, Addis Ababa. The clinic is providing service to Gondar and the neighboring residents both at the out-patient and in-patient level. There are 5 outpatient department rooms and 21 beds for the inpatients service. Currently, on per month average, up to 1200 psychiatric patients are attending their psychiatric follow-up service at the clinic.

### Inclusion and exclusion criteria

Patients’ ≥ 18 years of age and who were rated as having full insight based on the three-item insight measuring scale were included. Whereas, patents who were unable to communicate were excluded.

### Sample size determination and sampling procedure

The minimum number of samples required for the study was determined by using single population proportion formula considering the following assumptions:

$${\mathrm{n}}_{0}=\frac{{\mathrm{Z}}^{2}p\left(1-P\right)}{{\mathrm{d}}^{2}}$$

$${\mathrm{n}}_{0}=\frac{({1.96)}^{2}*0.321(1-0.321)}{{\mathrm{0,05}}^{2}}$$

$${\mathrm{n}}_{0} = 334.92 \approx 335$$

where: n_0_ = minimum sample size required for the study; Z = standard normal distribution (Z = 1.96) with confidence interval of 95% and ⍺ = 0.05; P = Hospital based prevalence of internalized stigma among PWMI (P = 32.1%) [30]; W = Absolute precision (W) = 0.05. Then, adding 10% (335 × 0.1 = 33.5 ≈ 34) of non-respondent rate, the total sample size for the study was 335 + 34 = 369. Participants of the study were selected using systematic random sampling technique. Respondents were screened to assess their eligibility for the study based on their insight status during the data collection time. Insight was measured using the three item insight measuring questions, where 1 represents “Yes” and 0 represents “No”. The total score ranges between zero and three. By adding the scores together, a score of zero, 1–2, and 3 were regarded as “no insight”, “partial insight”, and “full insight”, respectively. Those with full insight were selected as an eligible participants to be interviewed by the data collector [29]. Since the sample size of the study was decided to be 369, the sampling fraction (the K value) was obtained by dividing the total number of patients having service in 1 month (1200) for the total sample size (369)(1200/369 = 3.25 ≈ 3). Thus, the participants of the study were selected at regular interval (i.e. every 3rd) while the first participant was selected by lottery method.

### Study variables and data collection procedures

The dependent variable was internalized stigma which was measured using Internalized Stigma of Mental Illness (ISMI)-29 scale and dichotomized as high/low based on Ritsher et al. criteria [35]. While the independent variables were the patients’ socio-demographic variables (age, sex, residence, religion, educational status, marital status, and occupation), psychosocial factors (social support and self-esteem), substance related variables (current and life time use of substances such as chat, smoking, alcohol, etc.), clinical factors (types of psychiatric diagnosis, age at onset of mental illness, family history of mental illness, suicidal ideation, suicidal attempt, history of relapse, and history of hospitalization), and treatment factors (treatment duration, adherence to medication treatment, and psychotherapy service utilization). The variable psychotherapy service utilization was used to assess the current use of psychotherapy service by PWMI.

The data were collected by three trained psychiatry nurses by using a structured questionnaire (Additional file 1) which was prepared by reviewing similar studies [13, 17, 30, 33, 35–41] and the dependent variable internalized stigma was measured by using Internalized Stigma of Mental Illness (ISMI)-29 scale. The ISMI-29 scale was validated by another Ethiopian study which was conducted with the aim of determining the magnitude, domains, and covariates of internalized stigma among patients with mental illness in Dilla University Referral Hospital (DURH), South Ethiopia [30]. In the previous study in Ethiopia, the ISMI-29 scale had internal consistency reliability coefficient of alpha = 0.90. The ISMI-29 item scale has a whole of 29 items on a 4-point Likert scale (1 = strongly disagree, 2 = disagree, 3 = agree, and 4 = strongly agree). Each statement consisted of picks about a potential stigma issue. It incorporated five subscales; alienation (6 items), stereotype endorsement (7 items), discrimination experience (5 items), social withdrawal (6 items), and stigma resistance (5 items). Alienation is “the subjective experience of being less than a full member of society”. The Stereotype Endorsement is “the degree to which patients agreed with common stereotypes about people with a mental illness”. The Discrimination Experience measures “respondents’ perceptions of the way they tend to be treated by others”. The Social Withdrawal measures the self-exclusion from social events/situation due to mental illness”. The Stigma Resistance subscale is “a person’s ability to resist stigma” [20]. The stigma resistance subscale items were reverse coded (1 = strongly agree, 2 = agree, 3 = disagree, and 4 = strongly disagree); a higher score of stigma resistance indicating higher internalized stigma. In calculating the overall internalized stigma, it was considered as one subscale and it was summed together with the other four subscales. The overall internalized stigma score was obtained by summing the scores of the five subscales and divided by a total number of items. Finally based on Ritsher et al. criteria the overall internalized stigma of mental illness score was dichotomized as high/low (people with mental illness who scored between 2.51 and 4.00 were categorized as having higher internalized stigma while those who scored between 1.00 and 2.50 as having lower internalized stigma) [35]. Further, the questionnaire contains socio-demographic variables which shows patients’ background information and four standardized data collection instruments; ISMI-29 item scale [35], Oslo 3-item social support scale [41], Rosenberg Self-Esteem Scale [37], and Medication Adherence Rating Scale (MARS) [36].

Oslo 3-item social support scale was used to assess the social support. This scale has the sum score scale ranging from 3 to 14 with three broad categories: “poor support” 3–8, “moderate support” 9–11 and “strong support” 12–14 [41].

Rosenberg Self-Esteem Scale was used to assess the self-esteem of PWMI. The tool included 10-item questions with four viable response options which go from strongly agree “1” to strongly disagree “4” for each. On this scale, items 2,5,6,8, and 9 were reverse coded. Based on the sum score, the level of self-esteem of the study participants was categorized as low self-esteem (a sum score of < 15) and high self-esteem (a sum score of ≥ 15) [37].

Medication Adherence Rating Scale (MARS) that have ten items was used to measure participants’ medication taking behavior and attitudes. The total score of ≥ 6 indicates adherence and < 6 indicates non-adherence [36].

The current and life time substance use history was assessed using the items derived from Alcohol, Smoking and Substance Involvement Screening Test (ASSIST) [39] while suicidal ideation and attempt were assessed by using items taken from the Composite International Diagnostic Interview (CIDI) [38]. Data regarding socio-demographic, clinical, psychosocial, substance, and treatment related variables were collected through medical chart review and patients’ interview.

### Data quality control technique

Two weeks before the actual data collection a pilot study was carried out among 40 PWMI and modification was done on the questionnaire accordingly. Additionally, during data collection period, supervision was held regularly and the collected data were checked daily for completeness and consistence. Further, for handling missing data, it was entered by EPI DATA version 4.6.0.2, collected by interviewer administered questionnaire, incomplete data were excluded from analysis and only complete data were pass to final analysis.

### Data entry and statistical analysis

DAta was edited; cleaned, coded, entered into EPI DATA version 4.6.0.2, and exported to Statistical Package for Social Sciences version 20 for further analysis. Descriptive analysis was used to summarize the socio-demographic, clinical, psychosocial, substance, and treatment related characteristic of patients. Continuous variables were expressed as mean (± SD), while categorical variables were summarized as frequency (percentage). Binary logistic regression was used to determine factors associated with high internalized stigma. The Hosmer–Lemeshow goodness-of-fit test was used to assess the fitness of the model and a P-value was 0.65.

Both crude odds ratio (COR) and adjusted odds ratio (AOR) with the corresponding 95% confidence interval (CI) were calculated to show the strength of association. P value < 0.05 in the multi-variable regression model was considered as statistically significant.

### Operational definition

#### Mental Illness (MI)

Having disorders like schizophrenia, bipolar disorders, major depressive disorder, generalized anxiety disorder, and other diagnosable mental disorders [42].

#### Internalized stigma

Participants with a total score between 2.51 and 4 based on ISMI-29 scale were labeled as having high internalized stigma, while those with the score between 1.00 and 2.50 were labeled as having low internalized stigma [35].

#### Social support

Participants with a total score of 3–8, 9–11, 12–14 out of 14 based on OSLO-3 social support scale were labeled as having poor social support, moderate social support, and strong social support, respectively [41].

#### Self-esteem

Participants who scored ≥ 15 based on the Rosenberg Self-Esteem Scale total score were labeled as having higher self-esteem, while those with < 15 were labeled as having low self-esteem [37].

#### Current substance use

Consuming substance like chat, alcohol, tobacco, and others (cannabis, heroin) in the last 3 months [39].

#### Life time substance use

Consuming substance like chat, alcohol, tobacco, and others (cannabis, heroin) in the life time [39].

#### Suicidal ideation

Based on Composite International Diagnostic Interview (CIDI) as seriously thinking about committing suicide within the last month [38].

#### Suicidal attempt

Based on Composite International Diagnostic Interview (CIDI) as attempting to commit suicide within the last month [38].

## Result

### Socio-demographic characteristics of people with mental illness

A total of 365 people with mental illness were participated in this study, giving a response rate of 98.9%. The mean (± SD) age of the PWMI was 34.96 (± 11.26) years, 193 (52.9%) were males and 166 (45.5%) were married (Table 1).

**Table 1:** Socio-demographic characteristics of PWMI at UOGCSH, Northwest, Ethiopia, 2021

Variable	Category	Frequency (%)
Age	18–27 years	119 (32.6)
	28–37 years	109 (29.9)
	38–47 years	82 (22.5)
	48–57 years	40 (11.0)
	> 57 years	15 (4.1)
Gender	Male	193 (52.9)
	Female	172 (47.1)
Residence	Urban	172 (47.1)
	Rural	193 (52.9)
Religion	Orthodox	245 (67.1)
	Muslim	87 (23.8)
	Protestant	30 (8.2)
	Catholic	3 (0.8)
Marital status	Single	115 (31.5)
	Married	175 (47.9)
	Widowed	35 (9.6)
	Divorced	40 (11.0)
Level of education	Un able to read and write	92 (25.2)
	Primary	98 (26.8)
	Secondary	89 (24.4)
	College and above	86 (23.6)
Occupation	Unemployed	68 (18.6)
	Farmer	97 (26.6)
	Government employee	92 (25.2)
	Student	29 (7.9)
	Private employee	25 (6.8)
	Others	54 (14.8)*

%: percent

*Merchant, house wife, daily laborer, retired

### Clinical and treatment characteristics of people with mental illness

More than one-third, 147 (40.3%), of the PWMI were diagnosed with schizophrenia followed by bipolar disorder 84 (23.0). Majority of the PWMI, 223 (61.1%) and 268 (73.4%) had history of relapse, and hospital admission, respectively. Most of the PWMI, had no history of suicidal ideation 331 (90.7%) as well as suicidal attempt 345 (94.5%). Approximately one-fourth, 95 (26%), of the PWMI have got the psychotherapy service. Nearly two-third, 253 (69.3%), of the PWMI were non-adherent to their medication treatment (Table 2).

**Table 2:** Clinical and treatment characteristics of PWMI at UOGCSH, Northwest, Ethiopia, 2021

Variable	Category	Frequency (%)
Type of diagnosis	Schizophrenia	147 (40.3)
	Bipolar disorder	84 (23.0)
	Major depressive disorder	72 (19.7)
	Generalized anxiety disorder	38 (10.4)
	Others	24 (6.6)*
Age at onset of mental illness	≤ 18 years	28 (7.7)
	19–28 years	170 (46.6)
	29–38 years	88 (24.1)
	39–48 years	52 (14.2)
	49–58 years	21 (5.8)
	≥ 59 years	6 (1.6)
Suicidal ideation	Yes	34 (9.3)
	No	331 (90.7)
Suicidal attempt	Yes	20 (5.5)
	No	345 (94.5)
Family history of mental illness	Yes	99 (27.1)
	No	266 (72.9)
History of relapse	Yes	223 (61.1)
	No	142 (38.9)
History of hospital admission	Yes	268 (73.4)
	No	97 (26.6)
Duration of treatment	< 2 years	102 (27.9)
	2–5 years	141 (38.6)
	5–10 years	109 (29.9)
	> 10 years	13 (3.6)
Psychotherapy	Yes	95 (26.0)
	No	270 (74.0)
Reason of not getting psychotherapy	Distance	77 (21.1)
	No idea about psychotherapy	95 (26.0)
	Psychiatrist didn’t recommend me	85 (23.3)
	Don’t believe in psychotherapy	4 (1.1)
	Others	9 (2.5)**
Medication adherence	Adherent (≥ 6)	112 (30.7)
	Non adherent (< 6)	253 (69.3)

*Brief Psychotic disorder, sleep disorders, and post-partum psychosis

**Dissatisfied of my therapist, because I need it in private institution, tired of frequent appointment

### Psycho-social and substance use characteristics of people with mental illness

More than two-third, 321 (87.9%), of the PWMI had high self-esteem. Approximately half, 190 (52.1%), of the PWMI had moderate social support. In terms of life time substance use history, 123 (33.7%) of the PWMI were using a substance (Table 3).

**Table 3:** Psycho-social and substance use characteristics of PWMI at UOGCSH, Northwest, Ethiopia, 2021

Variable	Category	Frequency (%)
Self-esteem	High	321 (87.9)
	Low	44 (12.1)
Social-support		
	Poor	99 (2.1)
	Moderate	190 (52.1)
	Strong	76 (20.8)
Life time substance use history		
	Yes	123 (33.7)
	No	242 (66.3)
Current substance use		
	Yes	52 (14.2)
	No	313 (85.8)
Type of life time substance use		
	Alcohol	73 (20.0)
	Chat	17 (4.7)
	Tobacco	19 (5.2)
	Others	14 (3.8)*
Type of current substance use		
	Alcohol	24 (6.6)
	Chat	10 (2.7)
	Tobacco	12 (3.3)
	Others	6 (1.6)*

*Cannabis, heroin

### Prevalence of high internalized stigma

**Fig. 1:**
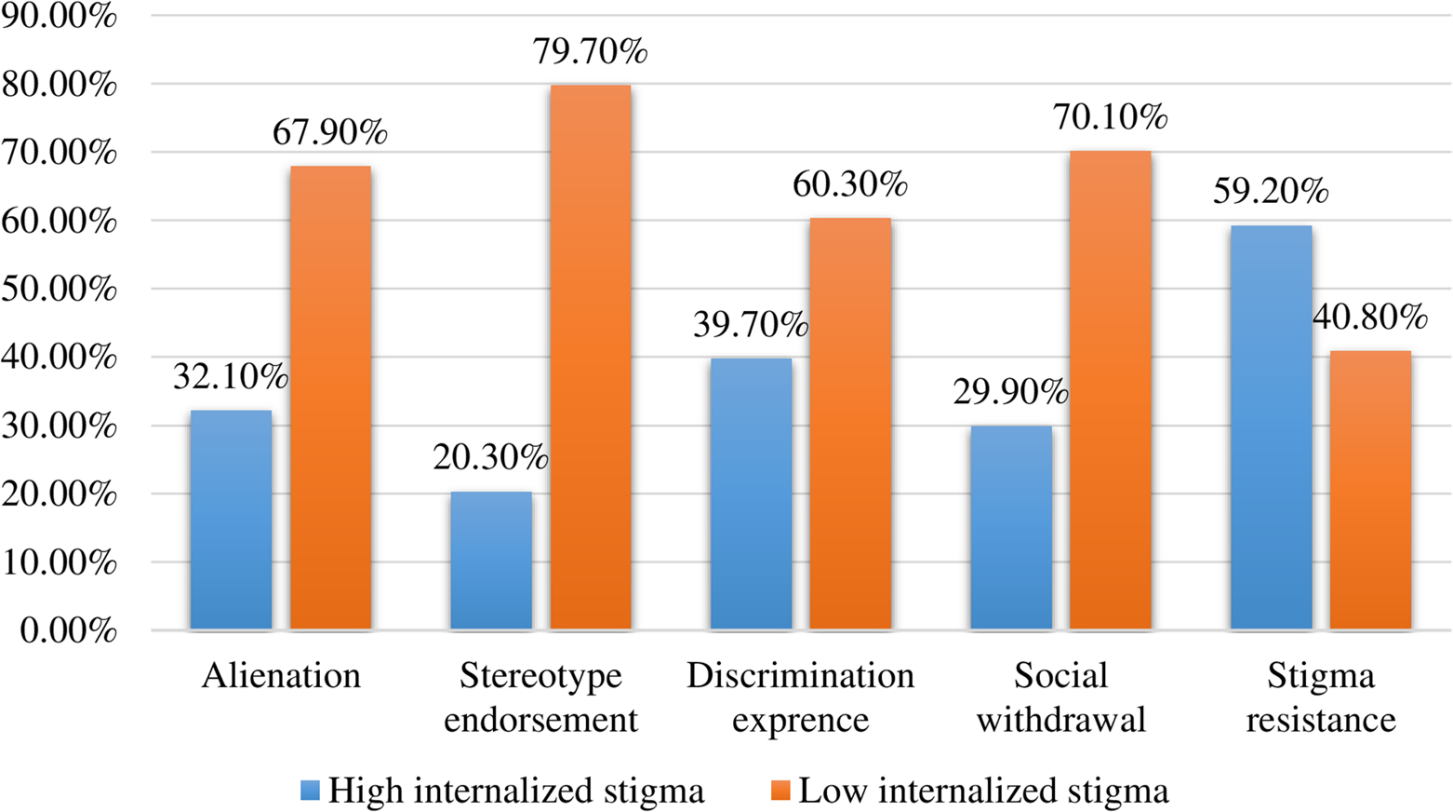
Internalized stigma with respect to domains among PWMI at UOGCSH, Northwest, Ethiopia, 2021

A high internal stigma was recorded in more than a quarter (27.9%; 95% CI 23.1–32.6) of PWMI. The prevalence of high internalized stigma with respect to domains was 32.1%, 20.3%, 39.7%, 29.9%, and 59.2% in alienation, stereotype endorsement, discrimination experience, social withdrawal, and stigma resistance, respectively (Fig. 1).

Regarding the distribution of high internalized stigma toward each item, 139 (38.1%) agreed with the item “Stereotypes about the mentally ill apply to me”, 180 (49.3%) agreed with the item “Living with mental illness has made me a tough survivor”, 175 (47.9%) were strongly disagreed with the item “Mentally ill people shouldn’t get married” (Table 4).

**Table 4:** Distribution of patients by their response to ISMI scale at UOGCSH, Northwest, Ethiopia, 2021

No	The 29 items of Internalized Stigma of Mental Illness (ISMI) scale	SD N (%)	D N (%)	A N (%)	SA N (%)
1	I feel out of place in the world because I have a mental illness	106 (29.0)	159 (43.6)	83 (22.7)	17 (4.7)
2	Having a mental illness has spoiled my life	79 (21.6)	132 (36.2)	118 (32.3)	36 (9.9)
3	People without mental illness could not possibly understand me	58 (15.9)	128 (35.1)	136 (37.3)	43 (11.8)
4	I am embarrassed or ashamed that I have a mental illness	79 (21.6)	163 (44.7)	101 (27.7)	22 (6.0)
5	I am disappointed in myself for having a mental illness	80 (21.6)	153 (41.9)	106 (29.0)	26 (7.1)
6	I feel inferior to others who don’t have a mental illness	86 (23.6)	131 (35.9)	103 (28.2)	45 (12.3)
7	Stereotypes about the mentally ill apply to me	68 (18.6)	74 (20.3)	139 (38.1)	84 (23.0)
8	People can tell that I have a mental illness by the way I look	141 (38.6)	102 (27.9)	94 (25.8)	28 (7.7)
9	Mentally ill people tend to be violent	170 (46.6)	125 (34.2)	54 (14.8)	16 (4.4)
10	Because I have a mental illness, I need others to make most decisions for me	157 (43.0)	123 (33.7)	71 (19.5)	14 (3.8)
11	People with mental illness cannot live a good, rewarding life	169 (46.3)	124 (34.0)	57 (15.6)	15 (4.1)
12	Mentally ill people shouldn’t get married	175 (47.9)	131 (35.9)	44 (12.1)	15 (4.1)
13	I can’t contribute anything to society because I have a mental illness	167 (45.8)	120 (32.9)	61 (16.7)	17 (4.7)
14	People discriminate against me because I have a mental illness	97 (26.6)	102 (27.9)	127 (34.8)	39 (10.7)
15	Others think that I can’t achieve much in life because I have a mental illness	101 (27.7)	105 (28.8)	124 (34.0)	35 (9.6)
16	People ignore me or take me less seriously just because I have a mental illness	108 (29.6)	111 (30.4)	113 (31.0)	33 (9.0)
17	People often patronize me, or treat me like a child, just because I have a mental illness	114 (31.2)	114 (31.2)	109 (29.9)	28 (7.7)
18	Nobody would be interested in getting close to me because I have a mental illness	126 (34.5)	123 (33.7)	94 (25.8)	22 (6.0)
19	I don’t talk about myself much because I don’t want to burden others with my mental illness	122 (33.4)	131 (35.9)	91 (24.9)	21 (5.8)
20	I don’t socialize as much as I used to because my mental illness might make me look or behave “weird.”	125 (34.2)	124 (34.0)	97 (26.6)	19 (5.2)
21	Negative stereotypes about mental illness keep me isolated from the “normal” world	129 (35.3)	117 (32.1)	96 (26.3)	23 (6.3)
22	I stay away from social situations in order to protect my family or friends from embarrassment	126 (34.5)	101 (27.7)	104 (28.5)	34 (9.3)
23	Being around people who don’t have a mental illness makes me feel out of place or inadequate	139 (38.1)	102 (27.9)	100 (27.4)	24 (6.6)
24	I avoid getting close to people who don’t have a mental illness to avoid rejection	146 (40.0)	101 (27.7)	99 (27.1)	19 (5.2)
25	I feel comfortable being seen in public with an obviously mentally ill person	136 (37.3)	89 (24.4)	107 (29.3)	33 (9.0)
26	People with mental illness make important contributions to society	72 (19.7)	98 (26.8)	152 (41.6)	43 (11.8)
27	Living with mental illness has made me a tough survivor	44 (12.1)	89 (24.4)	180 (49.3)	52 (14.2)
28	In general, I am able to live my life the way I want to	30 (8.2)	85 (23.3)	190 (52.1)	60 (16.4)
29	I can have a good, fulfilling life, despite my mental illness	45 (12.3)	72 (19.7)	189 (51.8)	59 (16.2)

### Factors associated with high internalized stigma

The factors significantly associated with high internalized stigma were male gender, occupation specifically; government employee, life time substance use, low self-esteem, and having previous history of hospitalization. Thus, the finding of the study suggested that male sex was 67% less likely to have higher internalized stigma compared to females (AOR = 0.33; 95%: 0.175–0.629). Compared to other group of occupation (merchant, house wife, daily laborer, retired), PWMI who were government employee were 69% less likely to have high internalized stigma (AOR = 0.31; 95% CI 0.118–0.809). PWMI who have history life time substance use were 3.56 times more likely have high internalized stigma compared to their counterparts (AOR = 3.56; 95% CI 1.867–6.793). Similarly, PWMI who have previous history of hospitalization were 4.24 times more likely to have high internalized stigma compared to their counterparts (AOR = 4.24; 95% CI 1.875, 9.570). Furthermore, PWMI who have low self-esteem were 8.31 times more likely to have high internalized stigma than those who have high self-esteem (AOR = 8.31; 95% CI 3.641–18.977) (Table 5).

**Table 5:** Bi-variable and multi-variable analysis of internalized stigma among PWMI at UOGCSH, Northwest, Ethiopia, 2021

Variable	Categories	Internalized stigma		COR (95% CI)	AOR (95% CI)
		High N	Low N		
Gender	Male	42	151	0.519 (0.326,0.826)	0.33 (0.175, 0.629) *
	Female	60	112	Ref	Ref
Occupation	Unemployed	30	38	1.579 (0.752, 3.313)	0.81 (0.328, 1.998)
	Farmer	26	71	0.732 (0.356, 1.508)	0.63 (0.263, 1.516)
	Government employee	13	79	0.329 (0.146,0.744)	0.31 (0.118, 0.809) *
	Student	8	21	0.762 (0.283, 2.054)	0.82 (0.257, 2.604)
	Private employee	7	18	0.778 (0.275, 2.201)	0.57 (0.167, 1.952)
	Others^#^	18	36	Ref	Ref
Diagnosis	Schizophrenia	54	93	2.206 (0.779, 6.246)	1.56 (0.455, 5.332)
	Major depressive disorder	19	53	1.362 (0.446, 4.158)	1.19 (0.308, 4.614)
	Bipolar	20	64	1.188 (0.393, 3.588)	0.84 (0.229, 3.074)
	Generalized anxiety disorder	4	34	0.447 (0.107, 1.867)	0.75 (0.126, 4.424)
	Others^##^	5	19	Ref	Ref
Life time substance use	Yes	44	79	1.767 (1.102, 2.833)	3.56 (1.867, 6.793)**
	No	58	184	Ref	Ref
Psychotherapy use	Yes	18	77	0.518 (0.292, 0.919)	0.51 (0.250, 1.029)
	No	84	186	Ref	Ref
Social support	Poor social support	42	57	2.063 (1.080, 3.943)	2.13 (0.978, 4.632)
	Moderate social support	40	150	0.747 (0.402, 1.386)	0.71 (0.350, 1.431)
	Strong social support	20	56	Ref	Ref
Medication adherence (MARS)	Adherent	23	89	0.569 (0.335,0.967)	0.56 (0.303, 1.026)
	Non-adherent	79	174	Ref	Ref
Self esteem	Low	26	18	4.656 (2.422, 8.953)	8.31 (3.641, 18.977)**
	High	76	245	Ref	Ref
History of hospitalization	Yes	90	178	3.581 (1.860, 6.898)	4.24 (1.875, 9.570)*
	No	12	85	Ref	Ref

^*^Statistically significant at p < 0.05, ** statistically significant at p < 0.01, Ref: Reference category; COR: Crude Odd Ratio; AOR: Adjusted Odd Ratio; CI: Confidence Interval; ^#^merchant, house wife, daily laborer, retired; ^##^brief Psychotic disorder, sleep disorders, and post-partum psychosis

## Discussion

In combating the negative influence of internalized stigma among PWMI, identification and targeting the potential risk factors for its high prevalence is the starting point. Through generating scientific evidence of risk factors of high internalized stigma, this study may provide data for clinicians on which factors they should target in reducing the internalized stigma of their patients, assist in designing and implementation of strategies which are essential for reducing internalized stigma among PWMI. This study showed that the prevalence of high internalized stigma among PWMI was 27.9% (95% CI 23.1, 32.6). This finding is consistent with previous studies conducted in Ethiopia specifically at Saint Paul’s Hospital (31.5%) [31], Dilla University Referral Hospital (32.1%) [30], and Jimma University Specialized Hospital (25.12%) [13].

However, it was lower than studies done in Nepal 54.44% [17], Singapore 43.6% [16], Iran 40% [20], and USA 36% [25]. This discrepancy might be due to difference in; socio-demographic as well as socio-culture of the study population, study setting, sample size, and cutoff points for ISMI score. The strong social bond observed in Ethiopian culture can protect the current study participants from developing higher percentage of internalized sigma. Further as evidenced in the current study majority of 266 (77.9%) the patients had good social support. Study in Singapore [16] was carried out in a large tertiary psychiatric care hospital where chronic and severe mental disorders that can increase the magnitude of internalized stigma are treated while the current study was done in psychiatric unit which was attached to UOGCSH. Comparing to the current study (n = 365), there were about 138, 280, 180, and 144 participants, in the study in Iran [20], Singapore [16], Nepal [17], and USA [25], respectively. The other variation might be taking of different cutoff points for ISMI score; study in USA [25] defined elevated internalized stigma as having a mean score of ≥ 1.5, whereas ≥ 2.51 in the current study.

On the other hand, the prevalence of the present study was higher than 8.1% in Korea [27], 17.5% in Serbia [26], and 9.8% in Qatar [19]. The possible reason for this discrepancy might be the use of various ISMI scales for assessing the levels of higher internalized stigma. For example, the study done in Qatar [19] used ISMI scale-18 items, unlike ISMI scale-29 items in the current study. The other reason might be due to a low level of awareness about mental illness among the Ethiopian population including the study participants of the current study. Additionally, it might be due to probability focusing of health care professionals on prescribing of medications, instead of providing strong psycho education, psychotherapy and counseling to prevent internalized stigma among psychiatric patients.

The study indicated that stigma resistance (59.2%) and discrimination experience (39.7%) were the two most common domains in which internalized stigma was highly prevalent. This was supported by other studies conducted in Ethiopia which reported that high internalized stigma was found in stigma resistance and discrimination experience domains [30, 31].

Comparing to males, females had higher internalized stigma which is similar with a study in Dilla University Referral Hospital [30] and Saint Paul’s Hospital [31], Ethiopia. This might be explained by females being exposed to a more blaming explanation of mental illness and social disadvantages additionally, it is believed that females are more stigmatized than males for the same behavior. They may have limited marriage invitation by males because of their illness. Therefore, such kinds of rejection may negatively influence internalized stigma.

Participants who were government employee were 69% likely to have high internalized stigma. This was similar with another study conducted in Ethiopia which reported that the prevalence of internalized stigma was higher among unemployed participants as compared to employed participants [33]. Other studies also showed that employment was significantly correlated with low level of internalized stigma and the odds of internalized stigma among unemployed were higher than employed participants [24, 43, 44]. A study conducted in United States of America explained that gainful employment has been found to reduce stigma towards PWMI [43].

PWMI who have low self-esteem were more likely to experience high internalized stigma compared with those who have high self-esteem. This is also supported by studies in Korea [27], in Singapore [16], Poland [45], Maryland [46], in Ethiopia; at Amanuel Mental Specialized Hospital [33] and Jimma University Specialized Hospital [13], Ethiopia. Evidences also showed that people with severe mental illness could have low self-esteem which reduces their ability to resist stigma [8, 47].

PWMI who had previous history of hospital admissions had higher internalized stigma than those who had no history of hospitalization. This finding was consistent with other study findings [15, 17, 33]. Repeated hospitalization in the past might show the severity and chronicity of a mental illness which may expose the PWMI to develop increased internalized stigma. Another possible explanation might be repeated absences from social situations due to frequent hospitalizations might make the patients easily stigmatized.

This study found that participants who had history life time substance use have higher internalized stigma than those who have not used substance in their lifetime. A study conducted in India also reported that substance use was significantly associated with high internalized stigma [28]. Further other studies also reported that there was a strong connection between substance use and internalized stigma [48, 49].

## Limitation of the study

Due to the cross-sectional nature of the study it cannot show the cause-effect relation between internalized stigma and significant associated factors. Since the study was conducted in a single centered health facility generalizability of the results might be limited.

## Conclusion

The results of this study showed that there was an intermediate prevalence of high internalized stigma among people with mental illness at University of Gondar Comprehensive Specialized Hospital. The hospital should promptly act to combat internalized stigma by giving emphasis to those who are female, have an occupation other than government employee, low self-esteem, history of life time substance use, and hospital admission.

## Supplementary Information

**Additional file 1.** Data collection tool.

## Data Availability

The datasets used/or analyzed during the current study is available from the corresponding author on reasonable request.

## Abbreviations

AOR: Adjusted Odd Ratio
CI: Confidence interval
COR: Crude Odd Ratio
ISMI: Internalized Stigma of Mental Illness
MI: Mental illness
PWMI: People with mental illness
UOGCSH: University of Gondar Comprehensive Specialized Hospital
