# Supplementary material for: Internalized stigma and associated factors among people with mental illness at University of Gondar Comprehensive Specialized Hospital, Northwest, Ethiopia, 2021
Source: Int J Ment Health Syst. 2022 Dec 31;16:58. doi: 10.1186/s13033-022-00567-2 (PMC9805673; doi:10.1186/s13033-022-00567-2)
Supplement: Supplementary file 1 — Additional file 1. Data collection tool. [file 13033_2022_567_MOESM1_ESM.docx]

**Questionnaire to assess the** **Internalized Stigma and Associated Factors Among People with Mental Illness at University of Gondar Comprehensive Specialized Hospital, Northwest, Ethiopia, 2021**

**Section I: socio-demographic characterestics of people with mental illness**

1. Age in years……………………….

2. Sex: A) Male B) Female

3. Residence: A) Urban B) Rural

4. Religion: A) Orthodox B) Muslim C) Protestant D) Others

5. Marita Status: A) Single B) Married C) Widowed D) Divorced

6. Level of eduaction:

A) Unable to write and read B) Primary eduaction (1-8) C) Secondary education (9-12) D) Collage and above.

7. Occupation:

A) Unemployed B) Farmer C) Governmental empolyee D) Student

E) Private employee F) Others (merchant, house wife, daily laborer, retired)

**Section II: clinical characteristics of people with mental illness**

1. Type of diagnosis

A) Schizophrenia B) Bipolar disorder C) Major depressive disorder

D) Generalized anxiety disorder E) Others (brief psychotic disorder, sleep disorders, and post-partum psychosis)

2. Age at onset of mental ilness (years) ……………………

3. Have you seriously thought about committing suicide within the last moth?

A) Yes B) No

4. Have you attempted to commit suicide within the last month? A) Yes B) No

5. Do you have a family member ever diagnosed with any of psychiatric disorders?

A)Yes B) No

6. Do you have hsitory of relapse? A) Yes B) No

7. Do you have history hospital admission? A) Yes B) No

**Section III: subtance use characteristics of people with mental illness**

1. In your life time, have you used any subtance ? A) Yes B) No

2. If your answer is yes for above question, then what kind of substance have you used?

1. Alchohol B) Chat C) Tobacco

D) Other (cannabis, heroin), specify ……………………..

3. In the past three months have you used any substances? A) Yes B) No

4. If your answer is yes for above question, then what kind of substance have you used?

1. Alchohol B) Chat C) Tobacco

D) Other (cannabis, heroin), specify ……………………..

**Section IV: treatment characteristics of people with mental illness**

1. For how long you have been on treatment for your mental illness (yesr)?...........................

3. Currently do you use a psychotherapy service as part of your treatment?

A) Yes B) No

4. If your answer is no for the above question, what do you think is the reason behind?

A) Distance B) I have no idea about psychotherapy C) My psychiatrist didn’t recommended me D) I don’t believe in psychotherapy treatment E) Other (dissatisfied of my therapist, because I need it in private institution, tired of frequent appointment)

5. People with mental illness response to Medication Adherence Rating Scale (MARS)

| S. no. | Question | Yes | No |
| --- | --- | --- | --- |
|  | Do you ever forget to take your medication? |  |  |
|  | Are you careless at times about taking your medication? |  |  |
|  | When you feel better, do you sometimes stop taking your medication? |  |  |
|  | Sometimes if you feel worse when you take the medication,  do you stop taking it? |  |  |
|  | I take my medication only when I am sick |  |  |
|  | It is unnatural for my mind and body to be controlled by medication |  |  |
|  | My thoughts are clearer on medication |  |  |
|  | By staying on medication, I can prevent getting sick. |  |  |
|  | I feel weird, like a ‘zombie’ on medication |  |  |
|  | Medication makes me feel tired and sluggish |  |  |

**Section V: psycho-social characteristics of people with mental illness**

Part A: people with mental illness response to Rosenberg Self-Esteem Scale.

Instruction: For each question below, please mark whether you strongly agree (1), agree (2), disagree (3), or strongly disagree (4).

| **No** | **Questions** | **Strongly agree** | **Agree** | **Disagree** | **Strongly disagree** |
| --- | --- | --- | --- | --- | --- |
| 1 | On the whole, I am satisfied with myself. | 1 | 2 | 3 | 4 |
| 2 | At times I think I am no good at all. | 1 | 2 | 3 | 4 |
| 3 | I feel that I have a number of good qualities. | 1 | 2 | 3 | 4 |
| 4 | I am able to do things as well as most other people. | 1 | 2 | 3 | 4 |
| 5 | I feel I do not have much to be proud of. | 1 | 2 | 3 | 4 |
| 6 | I certainly feel useless at times. | 1 | 2 | 3 | 4 |
| 7 | I feel that I'm a person of worth, at least on an equal plane with others. | 1 | 2 | 3 | 4 |
| 8 | I wish I could have more respect for myself. | 1 | 2 | 3 | 4 |
| 9 | All in all, I am inclined to feel that I am a failure. | 1 | 2 | 3 | 4 |
| 10 | I take a positive attitude toward myself. | 1 | 2 | 3 | 4 |
|  | Total Rosenberg Self-esteem Scale |  |  |  |  |

Part B: social support level of people with mental illness

1. How many people are you so close to that you can count on them if you have great personal problems?

A) None B) 1-2 C) 3-5 D) 6 and above

2. How much interest and concern do people show in what you do?

A) Very little B) Little C) Uncertain D) Some E) A lot

3. How easy is it to get practical help from neighbors if you should need it?

A) Very difficult B) Difficult C) Possible D) Easy E) Very easy

**SECTION VI: people with mental illness response to Internalized Stigma of Mental Illness (ISMI)-29 scale**

Instruction: For each question below, please mark whether you strongly disagree (1), disagree (2), agree (3), or strongly agree (4).

| **No** | **Questions** | **Strongly disagree** | **Disagree** | **Agree** | **Strongly agree** |
| --- | --- | --- | --- | --- | --- |
|  | **Alienation questions** | | | | |
| 1 | I feel out of place in the world because I have a mental illness. | 1 | 2 | 3 | 4 |
| 2 | Having a mental illness has spoiled my life. | 1 | 2 | 3 | 4 |
| 3 | People without mental illness could not possibly understand me. | 1 | 2 | 3 | 4 |
| 4 | I am embarrassed or ashamed that I have a mental illness. | 1 | 2 | 3 | 4 |
| 5 | I am disappointed in myself for having a mental illness. | 1 | 2 | 3 | 4 |
| 6 | I feel inferior to others who don’t have a mental illness. | 1 | 2 | 3 | 4 |
|  | **Stereotype endorsement questions** | | | | |
| 7 | Stereotypes about the mentally ill apply to me. | 1 | 2 | 3 | 4 |
| 8 | People can tell that I have a mental illness by the way I look. | 1 | 2 | 3 | 4 |
| 9 | Mentally ill people tend to be violent. | 1 | 2 | 3 | 4 |
| 10 | Because I have a mental illness, I need others to make most decisions for me. | 1 | 2 | 3 | 4 |
| 11 | People with mental illness cannot live a good, rewarding life. | 1 | 2 | 3 | 4 |
| 12 | Mentally ill people shouldn’t get married. | 1 | 2 | 3 | 4 |
| 13 | I can’t contribute anything to society because I have a mental illness. | 1 | 2 | 3 | 4 |
|  | **Discrimination experience questions** | | | | |
| 14 | People discriminate against me because I have a mental illness. | 1 | 2 | 3 | 4 |
| 15 | Others think that I can’t achieve much in life because I have a mental illness. | 1 | 2 | 3 | 4 |
| 16 | People ignore me or take me less seriously just because I have a mental illness. | 1 | 2 | 3 | 4 |
| 17 | People often patronize me, or treat me like a child, just because I have a mental illness. | 1 | 2 | 3 | 4 |
| 18 | Nobody would be interested in getting close to me because I have a mental illness. | 1 | 2 | 3 | 4 |
|  | **Social withdrawal questions** | | | | |
| 19 | I don’t talk about myself much because I don’t want to burden others with my mental illness. | 1 | 2 | 3 | 4 |
| 20 | I don’t socialize as much as I used to because my mental illness might make me look or behave “weird.” | 1 | 2 | 3 | 4 |
| 21 | Negative stereotypes about mental illness keep me isolated from the “normal” world. | 1 | 2 | 3 | 4 |
| 22 | I stay away from social situations in order to protect my family or friends from embarrassment. | 1 | 2 | 3 | 4 |
| 23 | Being around people who don’t have a mental illness makes me feel out of place or inadequate. | 1 | 2 | 3 | 4 |
| 24 | I avoid getting close to people who don’t have a mental illness to avoid rejection. | 1 | 2 | 3 | 4 |
|  | **Stigma resistance (reverse-coded items)** | **Strongly agree** | **Agree** | **Disagree** | **Strongly disagree** |
| 25 | I feel comfortable being seen in public with an obviously mentally ill person. | 1 | 2 | 3 | 4 |
| 26 | In general, I am able to live my life the way I want to. | 1 | 2 | 3 | 4 |
| 27 | I can have a good, fulfilling life, despite my mental illness. | 1 | 2 | 3 | 4 |
| 26 | People with mental illness make important contributions to society. | 1 | 2 | 3 | 4 |
| 27 | Living with mental illness has made me a tough survivor. | 1 | 2 | 3 | 4 |
|  | Total ISMI score | _________________________ | | | |
